# Heat Generation During Initial Osteotomy for Implant Site Preparation: An In Vitro Measurement Study

**DOI:** 10.1007/s12663-022-01800-8

**Published:** 2022-10-27

**Authors:** Luca Aquilanti, Luca Antognoli, Giorgio Rappelli, Roberto Di Felice, Lorenzo Scalise

**Affiliations:** 1 Department of Clinical Specialistic and Dental Sciences, Università Politecnica Delle Marche, Ancona, Italy; 2 Department of Industrial Engineering and Mathematical Sciences, Università Politecnica Delle Marche, Ancona, Italy; 3 Dentistry Clinic, National Institute of Health and Science of Aging, IRCCS INRCA, Ancona, Italy; 4 Private Practice, San Benedetto del Tronto, Italy

**Keywords:** Dental implant, Drill, Piezosurgery, Scanning electron microscopy, Heating

## Abstract

**Introduction:**

Controlling temperature generation during implant site preparation is important to prevent implant early failure.

**Aim:**

The present in vitro study aimed at measuring temperature variation generated during the initial osteotomy using both rotatory and piezo-surgical inserts.

**Methods:**

Nine groups were defined according to drill and insert type, cooling volume (mL/min) and cooling temperature. A total of 315 implant site preparations were performed in an artificial bone sample and the temperature was measured using an infrared camera. Drills’ wear was assessed using scanning electron microscopy at baseline and after 10 and 35 utilizations.

**Results:**

Piezo-surgical insert groups determined a temperature increase that was significantly higher than the one generated by rotatory drills groups (*p* < 0.001). When considering rotatory drills groups a temperature ≥ 40 °C was never recorded.

**Conclusion:**

Lower saline temperature implied a significant temperature decrease (*p* < 0.001), while the increase in cooling volume did not imply a temperature decrease. The scanning electron microscopy analysis of the drills demonstrated that little drill wear occurred up to 35 utilization times.

## Introduction

Bone is a complex organ that has been defined as a living organ due to its dynamic nature that allows the bone to participate in both mechanical and metabolic functions. Heat generated by implant site preparation could have negative sequalae for bone cell vitality, thus resulting in an increased risk of early implant failure. Thermal trauma is one of the causes that could lead to bone necrosis during surgical procedures and the extension of the necrosis is related to the amount of generated heat [1, 2]. Even though many cutoffs were proposed, generally, a temperature of 47 °C for 1 min is considered the critical threshold above which thermal necrosis occurs [3]. Similarly, bone damage could occur even with an excessive temperature decrease: A constant temperature of 3.5 °C was reported to determine a proven adverse effect on the bone and in the surrounding tissues [4, 5].

Many factors contribute to the increase of temperature during implant osteotomy and their control could enhance implant success rate. Generally, four different temperature conducive groups could be described: operator-dependent, manufacturer-dependent, site-dependent and patient-dependent [6]. The factors belonging to these categories could have both a direct and an indirect effect on thermogenesis, influencing each other and resulting in a temperature increase. Drill diameter, design and material, drill sharpness and wear, drill load, irrigation type, drilling procedure, drilling speed and time and bone density are some of the factors that determine a temperature generation during the osteotomy for dental implant placement [7]. In particular, a previous study demonstrated that the tiniest drill diameter corresponds to the highest temperature increase [8].

The management of heat generation during the initial osteotomy becomes very important in order to guarantee the safest clinical procedure and to control the factors that could have a role in implant early failure. The aim of the present in vitro study was to measure temperature increase generated during the initial implant site osteotomy using both rotatory and piezo-surgical inserts. The study aimed also at establishing the impact of the types of drills and insert in association with cooling volume and temperature on thermogenesis. Moreover, we evaluated drills wear and its influence on temperature increase.

## Materials and Methods

The present study measured temperature variations during the initial implant site preparation using an artificial bone sample. Twenty pounds per cubic foot (PCF) dense polyurethane blocks with 50 PCF dense 3 mm thick cortical plate were used for in vitro tests (Sawbones Europe AB, Malmö, Sweden). These blocks were chosen because they are able to simulate the human bone when considering drilling temperatures and drilling times [9].

**Fig. 1:**
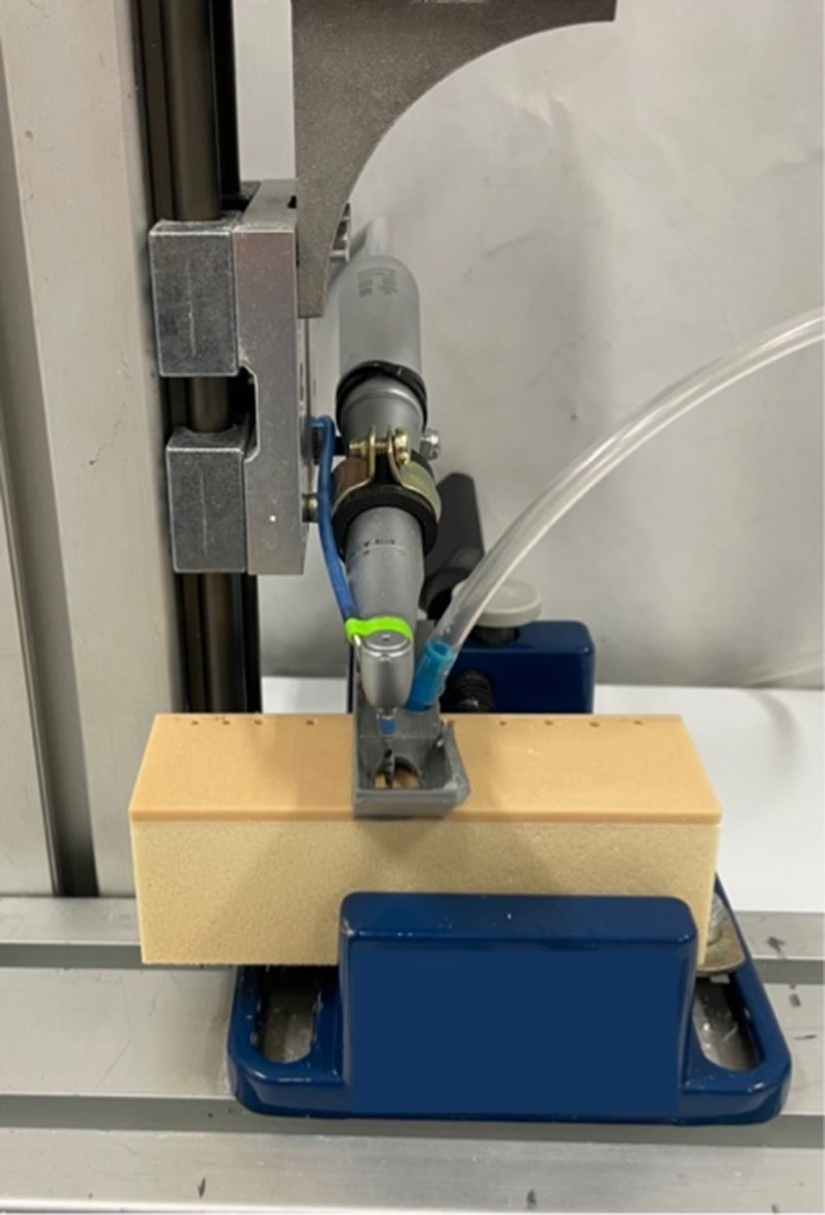
Customized test bench: detail of the sliding arm, implant handpiece and artificial bone block

Commercially available stainless steel pilot drills (Institute Straumann AG, Basel, Switzerland) and titanium nitride coated initial pilot osteotomy piezo-surgical insert (Mectron s.p.a., Carasco, Italy) were used. A customized test bench was designed and manufactured specifically for this study (Fig. 1).

Temperature was measured in °C by means of an infrared camera (Flir Systems, Inc., Oregon, USA). Thermal camera was installed at 30 cm from the artificial bone sample surface. Moreover, each drilling site was placed in the bone sample at a distance of 1.5 mm from thermal camera area of interest [10]. The camera detected infrared radiation from sample surface by the lens system and temperature variations were displayed as a color map. The nominal resolution of the camera was ± 0.01 °C and temperature ranges were from − 40 °C to 600 °C.

Nine groups were defined according to drill and insert type, cooling volume (mL/min) and cooling temperature:

- Group A (Standard Pilot Drill, 2.2 mm ⌀, single cooling system, saline temperature at 5 °C);
- Group B (Standard Pilot Drill, 2.2 mm ⌀, double cooling system, saline temperature at 5 °C);
- Group C (Standard Pilot Drill, 2.2 mm ⌀, single cooling system, saline temperature at 20 °C);
- Group D (Straumann X Pilot VeloDrill™, 2.2 mm ⌀, single cooling system, saline temperature at 5 °C);
- Group E (Straumann X Pilot VeloDrill™, 2.2 mm ⌀, double cooling system, saline temperature at 5 °C);
- Group F (Straumann X Pilot VeloDrill™, 2.2 mm ⌀, single cooling system, saline temperature at 20 °C);
- Group G (IM1S, single cooling system, saline temperature at 5 °C);
- Group H (IM1S, double cooling system, saline temperature at 5 °C);
- Group I (IM1S, single cooling system, saline temperature at 20 °C).

All the measurements were taken at a constant room temperature (22 ± 1 °C) and repeated 35 times (*n* = 315). In groups A, C, D, F, G and I, constant saline cooling of 120 mL/min was applied. In groups B, E and H, an additional saline cooling system (flow rate 70 mL/min) was applied at a constant distance of 1.5 mm from the drilling site. A surgical suction was applied at 2 cm from implant preparation site. Implant site preparations were performed reproducing the actual clinical situation and in accordance with the manufacturers recommended surgical techniques. The final drilling depth was set at 9 mm, and it was controlled by a vertical perforation stop. A vertical intermittent drilling procedure was used for all the tests (5 s drilling + 3 s withdrawing with continuous irrigation), setting drill rotational speed at 800 rpm for rotatory drills and “special” piezo-setup for piezo-surgical insert. A standard load of 9.8 N was applied on the handpiece, as reported elsewhere [7]. The rationale for applying the same load for all the groups was to measure the heat generated by the different study groups under the same test conditions. In Group I, a continuous vertical intermittent drilling procedure (1 s drilling + 1 s withdrawing with continuous irrigation) was performed as the preliminary results showed an excessive temperature increase in Groups G and H, probably due to the drilling procedure.

Drills’ wear was assessed using scanning electron microscopy (SEM) (Tescan Vega-3 LMU, Tescan Analytics, France) at baseline and after 10 and 35 utilizations. Surface cutting edge degradation was evaluated in terms of influence of wear on heat generation. Drills were not sterilized, but only rinsed with saline and dried with compressed air jets. Heat sterilization processes were not performed as they were reported to have no effect on temperature changes during implant site preparation [11].

Real-time thermographic videos were analyzed using MATLAB R2021b software (MatLab, MathWorks, Natick, MA, USA). MATLAB was used to register the temperature variations during each drilling procedure. Two regions of interests (ROI) at 4.5- and 9-mm depth were determined and both maximum temperature increase, and temperature variation were assessed. Data were analyzed using R statistical software (R Foundation for Statistical Computing, Vienna, Austria). Descriptive statistics were performed measuring temperature and temperature variation, outlining mean and standard deviation. The Kolmorov–Smirnov test was used to verify if data were normally distributed. A one-way ANOVA with Tukey post hoc test was used to assess significant differences among the groups. The correlation between temperature and the number of drills utilizations was calculated by Spearman’s rank correlation coefficient. The level of statistical significance was set at *α* = 0.05.

## Results

The temperature variations occurred during the initial implant site preparation were examined. Drilling insert type, drilling depth, saline volume and saline temperature were the variables which were considered. The mean artificial bone sample baseline temperature was 22.6 ± 1.9 °C in both the ROI.

**Fig. 2:**
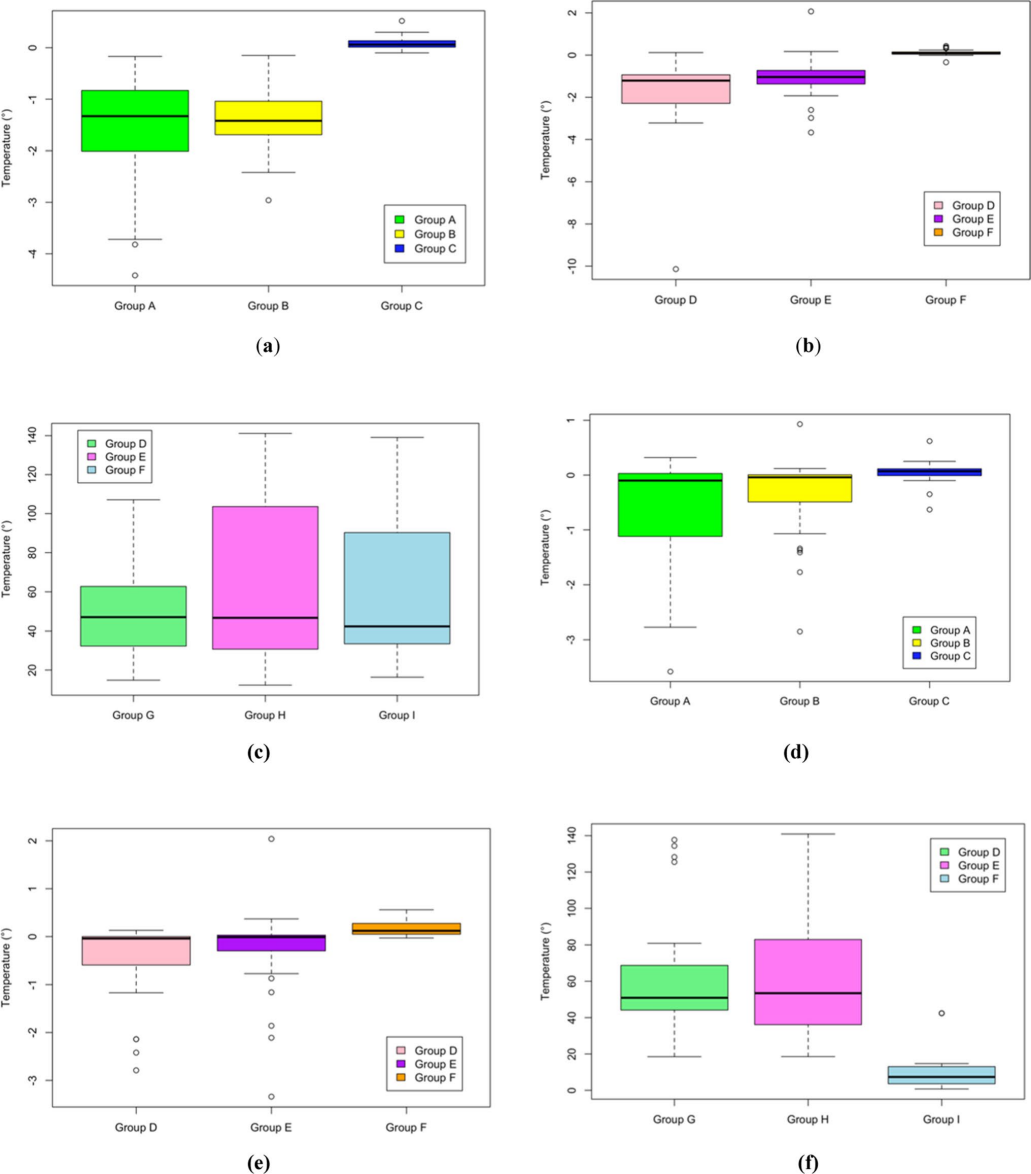
In this figure, boxplots of the recorded temperature variations (ΔT), in °C, are showed, accordingly to the different study groups and drilling depth (4.5 and 9 mm). In particular: **a** shows ΔT at 4.5 mm in Groups A, B and C; **b** shows ΔT at 4.5 mm in Groups D, E and F; **c** shows ΔT at 4.5 mm in Groups G, H and I; **d** shows ΔT at 9 mm in Groups A, B and C; **e** shows ΔT at 9 mm in Groups D, E and F; **f** shows ΔT at 9 mm in Groups G, H and I

Overall, both the maximum recorded temperature (MT) and the highest temperature variation (ΔT) were observed in Group H (*p* < 0.001) with a mean value of 87.27 ± 38.42 °C and 64.59 ± 38.37 °C, respectively. Conversely, Group C recorded the minimum temperature increase or a decrease in temperature itself and Group A the smallest ΔT (p < 0.001), 19.33 ± 0.07 °C and − 1.09 ± 0.91 °C, respectively (Fig. 2).

Table 1 shows the results derived from the analysis of temperature recorded during the 35-fold drillings. Generally, considering the same cooling saline temperature, it was not observed a statistically significant difference between the single and the double cooling system (*p* > 0.05). Conversely, temperature differed significantly when comparing the cooled saline with the not cooled one (*p* < 0.01). When considering Groups A, B, C, D, E and F, a temperature ≥ 40 °C was never recorded.

**Table 1:** Maximum recorded temperature and temperature variation, expressed in °C, recorded by the Thermal Camera at 4.5- and 9-mm depth in the 9 study groups

Temperature (°C)	Group B	Group B	Group C	Group D	Group E	Group F	Group G	Group H	Group I
MT^1^ 4.5 mm	21.02 ± 2.25	21.12 ± 1.27	19.58 ± 1.11	21.26 ± 1.26	22.20 ± 1.18	19.55 ± 1.19	75.39 ± 30.23	86.26 ± 41.81	84.10 ± 40.98 **
MT^1^ 9 mm	21.63 ± 2.19	21.63 ± 1.20	19.07 ± 1.23	21.76 ± 1.11	22.72 ± 1.09	19.12 ± 1.31	82.23 ± 30.56	88.27 ± 35.77	33.10 ± 12.18 **
ΔT^2^ 4.5 mm	− 1.54 ± 1.01 *	− 1.37 ± 0.58 **	0.90 ± 0.11	− 1.72 ± 1.67 **	− 1.10 ± 0.91 **	0.11 ± 0.14	53.35 ± 29.82	63.75 ± 41.76	62.79 ± 41.20 **
ΔT^2^ 9 mm	− 0.64 ± 0.96 *	− 0.33 ± 0.71 **	0.05 ± 0.19	− 0.42 ± 0.79 **	− 0.26 ± 0.86 **	0.17 ± 0.16	59.83 ± 30.28	65.42 ± 35.76	10.89 ± 12.29 **

Values are expressed as mean ± SD of results recorded during the 35-fold drillings

^1^Maximum recorded Temperature (MT); ^2^ Temperature Variation (ΔT)

* *p* < 0.01; ** *p* < 0.001

According to Spearman’s correlation, in Group A, strong correlations between ΔT at 9 mm and drill utilization times and MT at 9 mm and drill utilization times were reported (*rho* = 0.58, *p* < 0.001 and *rho* = 0.54, *p* < 0.01, respectively), while weak ones were observed between ΔT at 4.5 mm and drill utilization times and MT at 4.5 mm and drill utilization times (*rho* = 0.34, *p* < 0.05 and *rho* = 0.35, *p* < 0.05, respectively). In Group B, a strong correlation was only observed between ΔT at 9 mm and drill utilization times, resulting in a *rho* = 0.54, *p* < 0.001. In Group C, MT at 9 mm and drill utilization times were weakly correlated (*rho* = 0.34, *p* < 0.05). When considering Group D, a strong and a weak correlation were observed, respectively, for ΔT at 9 mm and drill uses (*rho* = 0.46, *p* < 0.01) and for MT at 9 mm and drill uses (*rho* = 0.37, *p* < 0.05). In Group E, ΔT at 9 mm and drill utilizations were strongly correlated, *rho* = 0.47, *p* < 0.01. In Group F, a weak correlation was detected between MT at 4.5 mm and drill usage (*rho* = 0.36, *p* < 0.05). When considering Groups G, H and I no correlations were observed among the studied variables (*p* > 0.05).

### SEM analysis

**Fig. 3:**
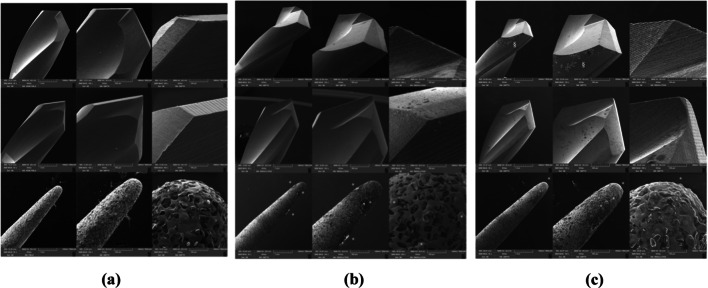
**a** Scanning electron microscope analysis of the used drills at baseline. No signs of wear were detected; **b** Scanning electron microscope analysis of the used drills after 10 uses. No signs of wear were detected on the cutting edges of rotary drills. Little presence of smear layer on the top of piezo-insert (*); **c** Scanning electron microscope analysis of the used drills after 35 uses. No signs of wear were detected on the cutting edges of rotary drills. Moreover, it was possible to notice the presence of water trees on the superficies of rotatory drills (§). Presence of smear layer on the top of piezo-insert (*)

Drills’ wear was assessed using SEM at baseline (Fig. 3a) and after 10 (Fig. 3b) and 35 utilizations (Fig. 3c).

Surface cutting edge degradation was evaluated in terms of influence of wear on heat genesis. As shown in Fig. 3, analyses were carried out using × 50, × 100 and × 500 magnification. Images were visually evaluated. No superficial differences were noticed among the drills at baseline and after 10 utilizations, apart from the little presence of a sort of smear layer on the top of piezo-inserts. The backscattered electron (BSE) detection showed the presence of elements with different atomic weight. Subsequentially, the energy dispersive X-ray analysis (EDX) was performed on the obtained images and apart from the chemical elements which compose steel (Iron, Manganese, Chromium, Silicon, Nickel, Aluminum, Sulfur, Nitrogen and Carbon), the presence of organic substance was revealed (Carbon, Phosphorus and Chlorine). The latter was related to the artificial bone block.

Analyzing drills’ surfaces after 35 utilizations, no signs of wear were detected on rotatory drills groups. When considering piezo-surgical insert groups, a bigger amount of smear layer was noticed, especially on the top of the insert. The BSE detection and the EDX analysis confirmed the presence of the organic substance that could be related to the artificial bone block.

## Discussion

A total of 315 initial implant site preparation osteotomies were performed to assess heat variation and generation during the initial drilling procedure, using an artificial bone sample. The utilization of a standard bone specimens is important for a rigorous comparison among tests. In fact, animal bone samples could lead dissimilarities due to different bone density and thickness between the cortical and the cancellous layer and among different samples. According to a previous study, the bone samples used in the present study are able to simulate human bones in terms of both drilling temperature and drilling times [9].

Overall, piezo-surgical insert groups determined a temperature increase that was significantly higher than the one generated by rotatory drills. The generated heat exceeded the limit threshold, thus, clinically, causing a possible damage to the bone cells, whose vitality is crucial for healing and osseointegration. Our results agree with those of previous papers reporting a significative temperature increase when comparing piezoelectric device with conventional drilling [12, 13]. The initial implant site preparation using piezo-inserts may determine a dangerous temperature increase. However, the protocol of the present study could have strained the results of the reported tests. In fact, in this study a load of 9.8 N was applied for all the tests: This load was probably too high for Groups G, H and I, as a maximum loading range from 100 to 400 g was suggested for piezoelectric surgery [14]. Moreover, Parmar et al. demonstrated that the type of the surface being cut significantly alter how the piezo-surgical tip oscillates. In particular, higher loading results in a modification of oscillatory shape which diminishes the amplitude of oscillation and the depth of cut [15]. Also, the used artificial bone block could have influenced the results. Through the SEM analysis, a block-related organic layer was observed. This organic residue was placed among the diamonds that coat the tip of the insert, possibly affecting the cutting efficacy. Also, the movement of the piezo-surgical handpiece could have affected the results. In fact, the correct use of ultrasonic tips during implant site preparation requires a light pressure load in association with a quarter turn rotary movement that allow an enhanced bone cutting efficacy and the dissipation of potential energy [16]. Unfortunately, the used sliding arm was not able to replicate such rotatory movement. Previous studies have demonstrated that ultrasonic implant site preparation technique is a viable alternative to the traditional drilling techniques, in terms of marginal bone level loss and risk of implant failure [17]. Moreover, implant survival rate seems not to be influenced by implant site preparation technique, with a better stability achieved in piezo-surgical techniques rather than conventional ones [18]. Nevertheless, great attention should be addressed to the followed clinical protocol to limit the risk of complications occurrence, also in terms of heat generation.

Lower saline temperature implied a significant temperature decrease when comparing the temperature in the area of interest using cooled or not saline, as also reported elsewhere [19]. Nevertheless, the mean temperature increase was below the critical threshold using either refrigerated cooling solution or not refrigerated one. A cooling system with a saline at 5 °C should be used when preparing implant site, but practically the utilization of a cooling system at room temperature could be as safe and efficient as cooled one in preventing dangerous temperature increase. No statistically significant difference was detected between low (120 mL/min) and high saline volume (190 mL/min) external cooling system, suggesting that the increase in cooling volume does not imply a temperature decrease. This datum is consistent with a recent study aimed at evaluating the saline distribution during implant osteotomy using a computational fluid dynamics model. In particular, up to a saline volume of 60 mL/min, the amount of the fluid inside the implant bed is proportional to the saline volume but increasing the irrigation volume to 80 mL/min does not significantly influence the fluid distribution [20].

Drilling depth affects temperature generation [21–23]. Greater drilling depths were associated to higher and significative temperature differences in Groups A, B, D and E. Not surprisingly, a more consistent temperature decrease was obtained at 4.5 mm rather than at 9 mm. When considering Groups C and F, no statistically significative differences were noticed between temperature variation at 4.5 and 9 mm. These data could be explained by the fact that the cooled saline had a bigger refrigerating effect in the most superficial areas of the specimen than in the deepest ones. Within the limits of the present study, in Groups G and H no significant differences were showed between the two drilling depths according to temperature increase. Probably, the incorrect handpiece movement and the consequent excessive temperature increase concealed the effect of cooling system. In Group I, a significative difference was detected between both MT and ΔT at 4.5 and 9 mm. In this case, conversely from the others, the deepest site recorded a minor temperature increase than the most superficial one: The continuous intermitting movement might have allowed the saline to reach the bottom of the preparation and the tinier diameter of the tip might be able to oscillate efficiently. Conversely, the greater diameter of the top of the insert could have been hampered by the inadequate diameter of the preparation, resulting in a potential energy dissipation decrease and a temperature increase.

The SEM analysis of the drills demonstrated that little drill wear occurred up to 35 utilization times. The most valuable signs of wear were detected on the cutting edges of both the used initial implant site preparation drills. Moreover, statistical analysis showed significant correlations between drills uses and drilling depth in terms of temperature increase at 9 mm, suggesting that a major sharpness loss occurred on drills tip. Even if the temperature increased, in Groups A, B, C, D, E and F the critical temperature threshold was never overcome, indicating the safeness of both the drills also after 35 uses. The results of the present study are in accordance with those of previous ones, but drills did not undergo to sterilization cycles [24, 25]. Although the findings of a previous study demonstrated that autoclave sterilization determines a loss of sharpness of drills, this was not deemed to significantly increase drill temperatures due to re-utilizations [26].

## Conclusions

Within the limitations of the present study, it is possible to conclude that:

- The 20-PCF dense polyurethane blocks with 50PCF dense 3 mm thick cortical plate should be used for in vitro studies aimed at assessing the behavior of rotatory drills.
- The usage of refrigerated saline (5 °C) significantly reduces temperature increase, during the initial implant osteotomy. Clinically, the overcoming of the critical temperature threshold is not likely to happen even when using not cooled saline (20 °C).
- The utilization of an increased volume of refrigerating liquid is not responsible for a significant benefit in terms of temperature increase control.
- The re-usage of implant pilot drills up to 35 times is safe and does not determine a significant temperature increase.
- Practitioners should follow strict clinical protocols and the recommendations for the correct usage of piezo-surgical devices.

## Data Availability

The data sets generated and/or analyzed during the present study are available from the corresponding author on reasonable request.
